# Practical Points

**Published:** 1899-02-04

**Authors:** 


### PRACTICAL POINTS.

### THE MEDICAL STAFF AND THE MANAGING COMMITTEE.

"*A Correspondent" writes: My committee are anxious to know what the general rule is with regard to hon. medical officers to cottage hospitals who do not subscribe. Should they be elected on the committee, or, rather, should the whole, as a matter of right, hold a seat?*

The point raised is fully dealt with in "Cottage Hospitals," a book of which every cottage hospital secretary should have a copy, as it contains matters of great importance to hospital management. The rules of a cottage hospital should define all who are eligible to be elected on the committee of management. They should provide whether every candidate must be a governor or whether there should be any members elected *ex officio* during their term of office at the hospital. The practice in vogue at the present time differs widely. It may be either of the following: (1) The whole of the medical staff, or (2) selected representatives of such staff, elected by themselves, may be on the committee; or (3) the medical staff may constitute a medical committee, and none of them shall be on the committee of management. Instances of each of these various plans might be quoted, and the general question is dealt with in a leading article in The Hospital of January 21st.

"*A Correspondent" writes: It has hitherto been the practice for the honorary medical staff to be elected by the Governors. Many members of the committee are in favour of altering this rule by giving power to the General Committee (a body consisting of 12 elected members and 21 ex officio members) instead of to the Governors. Which mode of election do you consider most suitable?*

The point raised is one of interest and importance. In our view the plan adopted at the Queen's Hospital, Birmingham, and, we believe, at the General Hospital, as well as at most hospitals where the rules have been modernised, is undoubtedly the best. At the Queen's Hospital, at each annual meeting of the Governors, an Election Committee of 100 Governors is appointed, with whom the election of the hon. medical officers rests during the year of appointment. The Committee of Election consists of the whole of the General Committee, the hon. medical officers *ex officio*, and such a number of Governors elected by ballot as with a committee and *ex officio* members together makes up a total of 100. With a view of putting down canvassing in advance, and making the election perfectly fair for all the candidates, it is the practice at some hospitals not to ballot for the general Governors until a vacancy occurs. The names of the whole of the Governors are put into a ballot box and the ballot is then taken, the names being added of the *ex officio* and committee members, and a complete list given to each candidate at the same time. Whether this plan is adopted or whether the ballot takes place at each annual meeting is, of course, a detail, and the circumstances of each institution decides which of the two systems of ballot is best adapted to its requirements. In our judgment the Governors would be well advised to adopt the Birmingham system, of which an outline is given above.

